# A Photoluminescence Study of the Changes Induced in the Zinc White Pigment by Formation of Zinc Complexes

**DOI:** 10.3390/ma10040340

**Published:** 2017-03-25

**Authors:** Alessia Artesani, Francesca Gherardi, Austin Nevin, Gianluca Valentini, Daniela Comelli

**Affiliations:** 1 Physics Department, Politecnico di Milano, Piazza Leonardo da Vinci, 20133 Milano, Italy; gianluca.valentini@polimi.it (G.V.); daniela.comelli@polimi.it (D.C.); 2 Chemistry, Materials and Chemical Engineering “Giulio Natta” Department, Politecnico di Milano, Piazza Leonardo da Vinci, 20133 Milano, Italy; francesca.gherardi@polimi.it; 3 Istituto di Fotonica e Nanotecnologie-Consiglio Nazionale delle Ricerche (IFN-CNR), Piazza Leonardo da Vinci, 20133 Milano, Italy; austin.nevin@ifn.cnr.it

**Keywords:** time-resolved photoluminescence, zinc white, zinc carboxylate, metal soap

## Abstract

It is known that oil paintings containing zinc white are subject to rapid degradation. This is caused by the interaction between the active groups of binder and the metal ions of the pigment, which gives rise to the formation of new zinc complexes (metal soaps). Ongoing studies on zinc white paints have been limited to the chemical mechanisms that lead to the formation of zinc complexes. On the contrary, little is known of the photo-physical changes induced in the zinc oxide crystal structure following this interaction. Time-resolved photoluminescence spectroscopy has been applied to follow modifications in the luminescent zinc white pigment when mixed with binder. Significant changes in trap state photoluminescence emissions have been detected: the enhancement of a blue emission combined with a change of the decay kinetic of the well-known green emission. Complementary data from molecular analysis of paints using Fourier transform infrared spectroscopy confirms the formation of zinc carboxylates and corroborates the mechanism for zinc complexes formation. We support the hypothesis that zinc ions migrate into binder creating novel vacancies, affecting the photoluminescence intensity and lifetime properties of zinc oxide. Here, we further demonstrate the advantages of a time-resolved photoluminescence approach for studying defects in semiconductor pigments.

## 1. Introduction

Time-resolved photoluminescence (TRPL) spectroscopy is a valuable method for material characterization that can be used to probe the spectral and kinetic properties of optical emissions [1]. Typically achieved at picosecond and nanosecond temporal resolution with the aid of fast detectors, TRPL is mainly used for studies of fast charge carrier dynamics with important applications in solar cells [2], quantum wells [3] and nanostructures [4,5]. In this work, TRPL is proposed for detecting the nanosecond and microsecond emission properties from band-to-band and trap state recombination paths in white paints containing zinc oxide (ZnO).

A new white pigment based on zinc oxide was introduced at the beginning of the nineteenth century in France [6]. Its diffusion was fostered mainly thanks to a publication by the paint manufacturers Winsor & Newton in 1837 [7] in which its superiority over lead white was claimed. In fact, zinc white is non-toxic, has good brilliance and can be easily mixed with other pigments, which favoured its production. Despite its many advantages, zinc white has also significant drawbacks as a paint material. Areas painted in white showed rapid degradation phenomena on their surface: localised cracking, yellowing and the formation of aggregates, sometimes visible to the naked eye, were reported as early as 1941 [8]. Due to its widespread use in late 19th and early 20th century paintings, great effort has been made in the understanding of degradation mechanisms that occur in painted layers.

Recent studies have shown that zinc white is highly reactive when mixed with oils and resins [9,10]. This is caused by the reactions between zinc ions of the pigment and fatty acids contained in the binding medium, leading to formation of zinc carboxylates, which are easily detected by Fourier transform infrared spectroscopy (FTIR) [11,12]. Clementi et al. worked on different mixtures of zinc white with oils and alkyd medium, observing the generation of a broad band at about 1600 cm^−1^ with infrared spectroscopy [13]. Based on literature about ZnO functionalized with organic network, the authors suggested that changes in luminescence were due to the absorption of free fatty acids onto the ZnO surface (chemisorbed carboxylates) and oxygen vacancies passivation. More recently, the chemical modification of painting layers has been investigated in depth. Through attenuated total reflection FTIR analysis of zinc paints, Hermans et al. [14] have demonstrated that carboxylates chemisorbed on the ZnO surface represent only a minor fraction of the metal carboxylate species. The authors stated that, most likely, metal ions diffuse in the binding medium and then are bound to carboxylate groups. Thereafter, these aggregates increase in size forming metal soaps and migrate to the painting surface [15,16].

In order to provide a complete view of the formation mechanisms of carboxylates in paints, a complementary approach is here proposed, based on the study of photo-physical changes induced in the ZnO crystal structure following pigment–binder interaction. In general, ZnO as a semiconductor material shows two characteristic optical emissions. The first is the direct band-to-band recombination (BE) that produces a narrow emission at 380 nm. The second characteristic emission is from trap state levels (TS), with the most intense and well-known emission being centred in the visible spectral region (around 530 nm). The origin of the green emission is widely discussed in literature [17,18] and the most recent studies lead to the hypothesis that it originates from various types of ZnO surface intrinsic defects [19,20].

In our previous paper, significant differences from the expected PL behaviour of ZnO were reported in historical pastels, produced by embedding ZnO in an organic matrix [21]. In this work, we perform a systematic study to compare the photoluminescence (PL) properties of zinc white pigment in powder and the same mixed with different reactive and non-reactive organic binders in paint. The study aims to provide new insights into the link between zinc complex formation and luminescence properties. For the purpose of characterizing the relationship between ZnO luminescence and complex formation, in this work TRPL analysis is complemented by Fourier transform infrared (FTIR) spectroscopy in order to detect chemical changes in the prepared paint samples and specifically the formation of metal carboxylates.

## 2. Materials and Methods

### 2.1. Sample Preparation

In order to study the binder effect on photoluminescence emission of zinc white, paint mock-ups and reference analytical grade samples were studied. Paint mock-ups were prepared by mixing zinc white powder (46300, Kremer Pigmente GmbH & Co. KG, Aichstetten, Germany) and analytical grade zinc oxide (99% pure Sigma Aldrich, Milano, Italy) with three different binders each. Specifically, stand linseed oil (3320, Zecchi, Firenze, Italy) and chios mastic varnish in turpentine (3510, Zecchi, Firenze, Italy) were mixed with zinc white (1:1 in weight for each paint). A third paint was obtained by mixing 1 part in volume of zinc white, 1 part of gum arabic powder (2310, Zecchi, Firenze, Italy) and 2 parts of distilled water. The same preparation was done with zinc oxide powder instead than zinc white, obtaining in this way six mock-up samples (Table 1). Following preparation, each painting material was homogeneously spread onto quartz discs (Corning(R) HPFS(R) 7980 fused silica) as a thick layer (1–2 mm). In addition, commercial zinc white, available as a premixed oil color (Rembrandt, 104 series), was employed to prepare a further paint mock up on quartz disc. The measurements described in the next section were performed at least seven days after sample preparation, in order to allow the surface to dry. During this time, the samples were kept in the dark and in normal environmental conditions.

As reference samples, analytical grade zinc oxide (99% pure Sigma Aldrich, Milano, Italy) and zinc white powder (Kremer Pigmente GmbH & Co. KG, Aichstetten, Germany) were selected and analyzed as powders, gently pressed in a sample holder. Moreover, the photoluminescence emissions from binding media alone, zinc stearate (307564, Sigma Aldrich, Milano, Italy) and zinc palmitate (CDS003313 A, Sigma Aldrich, Milano, Italy) were recorded to compare data with that from mock-up samples. All samples are listed in Table 1.

### 2.2. Methods

#### 2.2.1. FTIR Spectroscopy

Micro samples from painted layers were collected and analyzed by Fourier transform infrared spectroscopy (FTIR), using a Nicolet 6700 spectrophotometer coupled with Nicolet Continuum FTIR microscope equipped with an MCT detector (acquired between 4000 and 600 cm^−1^ with 128 acquisitions and 4 cm^−1^ resolution) using a micro compression diamond cell accessory. The spectra were baseline corrected using Omnic software. The FTIR spectra from stand linseed oil and mastic-based paints were normalized based on the intensity of the carbonyl C=O stretching vibration at approximately 1740 and 1710 cm^−1^ respectively. All other spectra were normalized to the ester carbonyl band at 1738 cm^−1^.

#### 2.2.2. TRPL Spectroscopy

The TRPL spectroscopy set-up has been described in detail elsewhere [21]. Here, its main features are briefly reported together with the measurement and analysis protocol adopted in this work. The system, based on pulsed excitation and time-gated detection, allows analysis of the PL emission from a circular spot of 1 mm in diameter on sample surface. Excitation of PL is provided by a Q-switching laser, emitting sub-ns pulses at 355 nm. PL emission is dispersed by an imaging spectrometer (covering the spectral range 380–750 nm) and then detected by a time-gated intensified camera with a gate width adjustable from 5 ns to continuous mode.

The measurements are based on the detection of a sequence of PL gated spectra at different delays with respect to laser pulses. In this work, short-lived and long-lived emission was detected by employing different gate widths.

1. Fast recombination emission was detected using a gate width of 5 ns and recording the emission decay kinetic for the first 50 ns following excitation. The spectrometer slit was set to 50 μm, giving rise to a spectral bandwidth of 2 nm.
2. Long-lived emission at the microsecond timescale was detected using a gate width of 1 μs and recording the decay kinetic for 100 μs following excitation. The fainter microsecond emission was detected by increasing the spectrometer slit to 200 μm, equivalent to a spectral bandwidth of 4 nm.

Following proper calibration of spectral data and correction for the detector efficiency, it is possible to observe the PL spectrum from the samples in a selected temporal gate window (in the following quoted as gated spectra) or to observe the emission decay kinetic in a proper spectral window. In the latter case, a multi-exponential decay (with a maximum of three components) was fitted to the kinetic data integrated over a selected spectral region [22] using a nonlinear least squares fitting methods applied to the model function:

(1) $$I\left(t\right)=\underset{i}{\sum }A_{i}\tau_{i}\left(1-e^{-w/\tau_{i}}\right)e^{-t/\tau_{i}}$$

where *t* is the time delay, *τ_i_* and *A_i_* are the lifetime and the intensity of the i-th decay component, respectively, and *w* is the temporal width of the experimental gate. The effective lifetime $\tau_{eff}$ is then calculated as the average of the lifetimes weighted over the number of photons originating from each decay path, according to the equation:

(2) $$\tau_{eff}=\frac{\Sigma_{i}A_{i}\tau_{i}^{2}}{\Sigma_{i}A_{i}\tau_{i}}$$

## 3. Results

FTIR and TRPL spectroscopy analyses of mock-up samples based on zinc white and pure zinc oxide have provided very similar results. Hence, in the following sections only data from mock-up samples based on zinc white are reported, whereas the whole set of data is provided in the Supplementary Materials.

### 3.1. FTIR Spectroscopy

**Figure 1:**
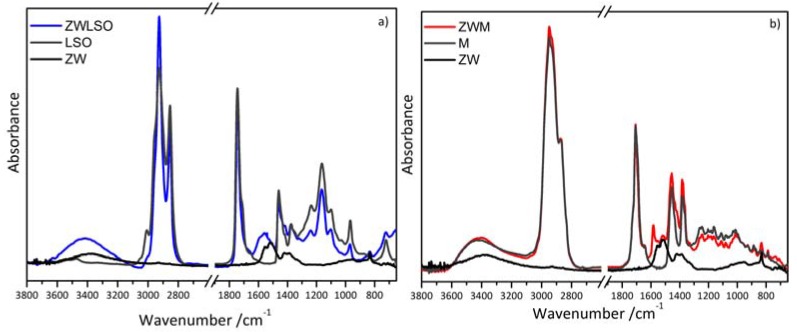

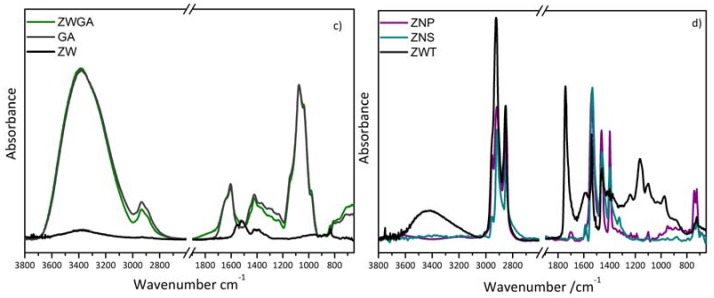
FTIR spectrum of (**a**) zinc white powder (ZW), linseed oil (LSO) and paint made of zinc white and stand linseed oil (ZWLSO); (**b**) zinc white powder (ZW), mastic (M) and paint made of zinc white and mastic (ZWM); (**c**) zinc white powder (ZW), gum arabic (GA) and paint made of zinc white and gum arabic (ZWGA); (**d**) commercial oil-based paint containing zinc white (ZWT), crystalline zinc stearate (ZWS) and zinc palmitate (ZWP) reference standard. Spectra are shown following background correction.

As reported in Figure S1 (Supplementary Materials), the FTIR spectrum of ZnO reference analytical grade does not present significant absorption bands in the medium infrared spectral range. The main absorption of ZnO occurs below 600 cm^−1^, which is out of the detection range of the sensor used in the employed FTIR spectrometer. On the contrary, the zinc white pigment (ZW) is zinc oxide with traces of zinc carbonate (Figure S1). FTIR spectra from mock-up samples (ZWLSO, ZWM, ZWGA) and the commercial zinc white (ZWT) are reported in Figure 1 and summarised in Table 2. Spectra show the expected absorption bands from their components, zinc white and binders, and the absorption bands ascribed to reaction products.

In detail, the FTIR spectrum of the ZWLSO sample (Figure 1a) is characterized by the bands ascribed to linseed oil (see Table 2). In addition, the broad band with a maximum at about 1550 cm^−1^ with a shoulder at 1598 cm^−1^ and the band at about 1410 cm^−1^ have been detected. They are related to asymmetric and symmetric COO– stretching vibrations, respectively. These bands indicate the presence of amorphous zinc carboxylates species, where zinc is coordinated to different carboxylate-based compounds [11]. Mastic resin is a complex system based on apolar terpenes, polar terpenoids and high molecular weight compounds, which have been fully characterized before and after accelerated ageing [23]. Zinc oxide in the ZWM sample, as observed in the linseed oil-based mock up, reacts with carboxylic groups of the triterpenic acids producing zinc salts of resin acids, as evidenced by the peak at about 1587 cm^−1^ in the related FTIR spectrum (Figure 1b). This peak is ascribed to the formation of zinc carboxylate complexes, in which zinc ions are bound to carboxylate groups of resin acids. Similar results have been documented for shellac and other terpenoids, which can react with lead, zinc, iron, manganese and copper ions, producing metal carboxylate compounds of resin acids [24,25]. As reported above for linseed oil-based paint, the formation of zinc salts in resin is very rapid and occurs during the first stages of the drying of the paint mock-up [26]. Gum arabic is a plant gum based on aldopentoses, aldohexoses and uronic acids [27] and its characteristic FTIR spectrum is shown in Figure 1c. In sample ZWGA, no significant differences between the spectra obtained from gum arabic and the paint can be observed. The broadening of the base of the band at 1600 cm^−1^ (Figure 1c) indicates a small interaction between gum arabic and the pigment.

A commercial oil-based paint containing zinc white was also studied in comparison with the mock ups. In addition to the broad band at 1585 cm^−1^, related to amorphous zinc carboxylates, a sharp band at about 1540 cm^−1^ can be observed and corresponds to a crystalline zinc soap from saturated fatty acids formed in the oil medium [28,29] (see Figure 1d and Table 2). The sharp band matches bands in the spectra obtained from crystalline Zn stearate and palmitate reference standards (Figure 1d) and could be due to inhomogeneous concentration of ‘free’ fatty acids in the formulations or to the addition of carboxylate-based salts as driers to promote and accelerate the curing of the binding oil medium [11,30].

### 3.2. Time-Resolved Photoluminescence Spectroscopy

**Figure 2:**
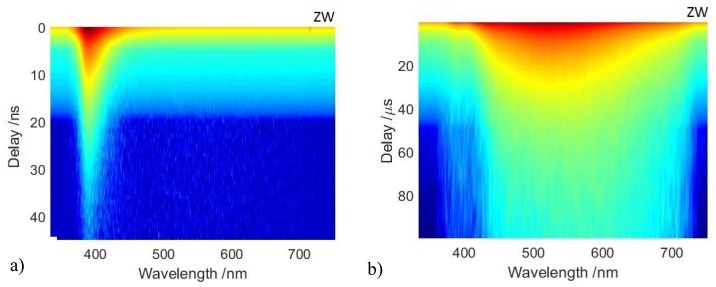

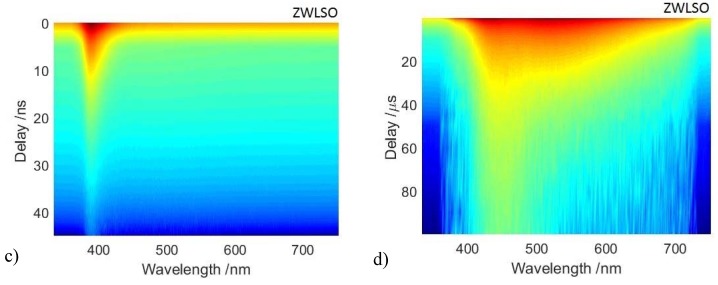
Two-dimensional time-resolved photoluminescence (TRPL) datasets, taken as a function of emission wavelength and delay time of gating detection respect to laser excitation for the pure zinc white pigment (ZW) (top) and for zinc white in linseed oil (ZWLSO) (bottom). The photoluminescence at nanosecond delays (**a**,**c**) and microsecond delays (**b**,**d**) allows the detection of BE and TS emissions, respectively.

TRPL analysis, performed at the nanosecond and microsecond timescales, allows the detection and differentiatiation of the BE and TS emissions in all analysed samples. An effective example of the benefits of the time-resolved approach is provided in Figure 2, where the two-dimensional TRPL datasets detected for the ZW and ZWLSO samples are shown. The TRPL dataset of all samples are reported in Supplementary Material (Figure S2, S3, S4). Nanosecond TRPL analysis shows that, in mock-up paint samples, the shape and peak position of direct recombination from near BE levels does not change with respect to zinc white powder (Supplementary Material). The effective lifetime changes with an average value of τeff = 1.5 ns (σ = ±0.2 ns), and the shortest effective lifetime is found for the zinc white powder pigment (τeff = 1.3 ns) and for pure zinc oxide (τeff = 1.0 ns) (Table S1 and Figure S5). This first result is in agreement with previously reported results from historical painting materials [21]. Microsecond TRPL analysis demonstrates two distinct emissions due to recombination via trap state levels. The emissions are detected in all ZnO based samples (as pure materials and paints): the first is an emission in the blue region, with a maximum emission at 430 nm (BL), whereas the second is the well-known broad emission in the green region centered around 530 nm (GL). In addition, it is observed that in all mock-up paint samples the interaction between ZnO and the binding medium gives rise to a net increase of the ratio between the TS and the BE emission with respect to that observed in the pure zinc white pigment (Table 3).

**Figure 3:**
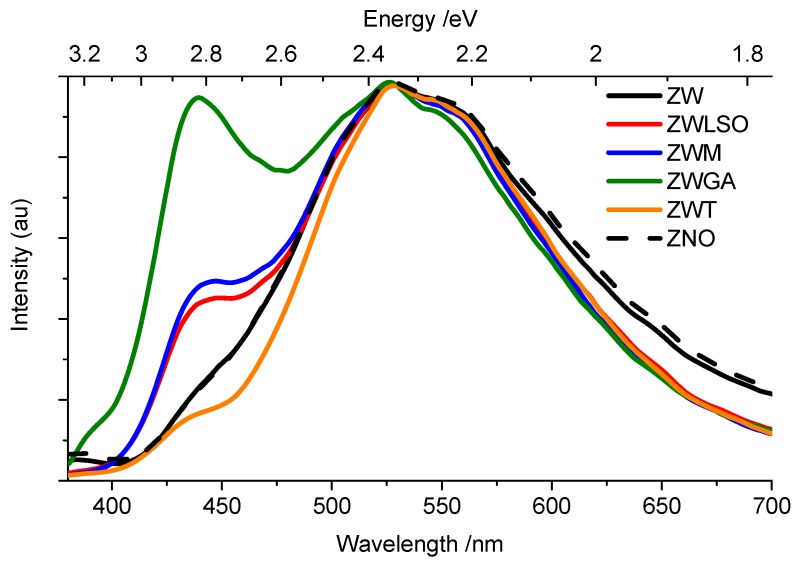
Photoluminescence (PL) gated spectra (time delay: 1–5 μs) of mock-up samples (ZWLSO, ZWM, ZWGA), commercial oil-based paint containing zinc white (ZWT), zinc white powder (ZW) and pure zinc oxide (ZNO). All spectra are normalized to the 530-nm peak and corrected for instrumental efficiency.

**Figure 4:**
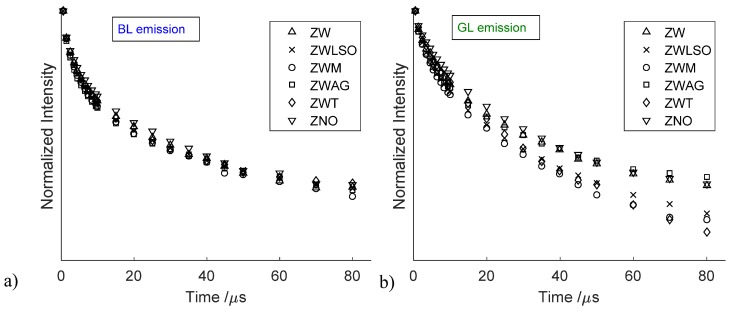
Emission intensity as a function of delay time respect to laser excitation. The delay value of 500 ns was set as time zero (**a**): decay kinetic of blue emission (BL at 430 nm); (**b**): decay kinetic of green emission (GL at 530 nm).

Detailed analysis of TS emissions is provided in Figure 3, where mock-up and pure ZnO samples are compared in terms of gated spectrum in the gate window 1–5 μs. The BL emission is undoubtedly stronger in mock-up paint samples, indeed it is still visible in the pure material as a left-shoulder. Despite differences in the relative intensity among samples, the BL emission decay kinetic (in the spectral band 410–450 nm) shows little differences among samples, with values ranging between τeff = 4.8–5.5 μs (Figure 4a). Details of time-resolved analysis are reported in Supplementary Material (Figure S6 and Table S2). In contrast, the GL emission has a net variability among samples in terms of emission decay kinetic. In particular, a longer effective lifetime was found in analytical grade zinc oxide, zinc white powder and in the mock-up sample based on gum arabic (τeff = 5.4–7.0 μs). A shorter effective lifetime was detected in painted samples prepared with linseed oil and mastic (τeff = 4.3 μs) and commercial zinc white paint (τeff = 5.0 μs) (Figure 4b). For the sake of completeness, the optical emissions of selected binders, zinc palmitate and zinc stearate are reported in Supplementary Material. Here, we stress that reference binding media (LSO, M, GA) mainly emits in the visible spectral region (400-600 nm) with a nanosecond emission decay kinetic. Similar emission properties are detected for the zinc stearate reference samples which contain unreacted fatty acids (see Table 2) (ZNS, ZNP).

## 4. Discussion

FTIR analysis of the samples examined in this work has demonstrated the presence of traces of zinc carbonate as well as the formation of various zinc complexes in paint and paint mock ups. It is important to underline that the microsecond PL emission detected from our paint samples is not attributed to these reaction products or to zinc carbonates, which instead are characterized by a nanosecond emission (see Supplementary Material Figure S7, S8). Indeed, microsecond PL emission is related to optical transitions via trap state levels in the ZnO-based pigment. Therefore, changes in this timescale have to be attributed to modifications of ZnO crystal defects induced by chemical interactions with the binder.

PL emission from near BE recombination of zinc oxide is only slightly affected by the presence of the surrounding organic binder principally in decay kinetic. Nonetheless, the spectral features of this emission are unaltered due to the high stability of the crystalline structure of the semiconductor pigment. For this reason, authors have suggested to use the characteristic emission at 380 nm for diagnostic purposes [12]. The slight increase in the BE emission decay lifetime in presence of a binding medium is ascribed to the superposition of the nanosecond emission from the binder, which partially overlaps the ZnO BE emission.

**Figure 5:**
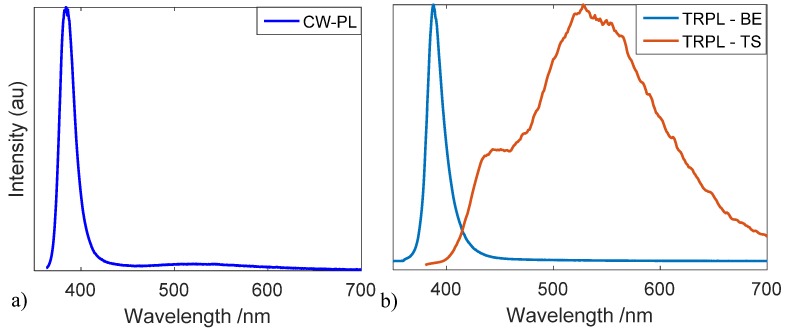
The photoluminescence emitted by zinc white sample mixed with linseed oil (ZWLSO) detected with (**a**) steady-state detector and (**b**) time-resolved detector. In the first case, the intensity of direct recombination prevents the detection of signal from deep-level emission. The problem is overcome by gating the PL emission with a time-resolved system.

Analysis of the PL emission in the microsecond timescale, related to recombination via trap state levels, provides more interesting data: (i) first, we observe that the PL intensity ratio of deep-level emission and direct band-to-band recombination increases in zinc white samples mixed with an organic binder with respect to the pure material. The ratio is often used to evaluate the concentrations of structural defects [31]. On this basis, we suggest that the chemical interaction of the semiconductor pigment with the surrounding organic medium leads to an increase of trap state density concentration; (ii)second, thanks to a time-gated detection technique, the overlap with binder fluorescence can be avoided and the presence of peculiar emissions from trap state levels can be easily detected. In particular, we detect the presence of a TS emission in the blue spectral region in all ZnO samples, an emission which is little observed and discussed in the literature for bulk ZnO-based samples. To the authors’ knowledge, a similar PL emission-peaked around 410–430 nm was identified with the aid of synchrotron-PL micro-imaging [32,33] in isolated microscopic areas of zinc white painted samples. The authors suggested that that specific emission could be related to a distinct crystal defect within ZnO crystals, possibly indicative of long-term alteration of pigment material. Indeed, beside this photo-physical interpretation, it is here noted that the use of common detectors working in continuous mode do not allow the easy detection of the faint BL emission from trap state levels due to partial spectral superposition with the much more intense direct BE recombination emission. An illustrative example of the benefits provided by employing a time-gated detection approach with respect to a conventional continuous mode set-up is provided in Figure 5.

Beside ZnO bulk materials, a PL emission centered around 420–440 nm was previously detected in ZnO nanoparticles [34,35,36], whose origin has been tentatively assigned to electron transition from shallow donor level of neutral Zn interstitial to the top valence band level [34]. A clear understanding of the nature of crystal defects ascribed to the BL optical emission in our samples is out of the scope of the present work and it could be achieved only with further experimental research and proper first-principles calculations of ZnO density states. Here, on the basis of experimental data, we provide some hypotheses: the relative intensity of the BL emission is enhanced in ZnO samples embedded in an organic binder, as seen in paints prepared with gum arabic, without a noticeable change in the emission lifetime. We can hence state that the pigment–binder chemical interaction simply promotes the formation of a larger number of a specific type of crystal defects. Considering that the common features of the selected binders is the presence of hydroxyl groups (OH), which is particularly abundant in gum arabic, we hypothesise a complexation of the hydroxyl group, which gives rise to the promotion of specific types of ZnO defects.

In parallel, the well-known green emission (530 nm) [17,18,19,37] shows different decay kinetic behaviour depending on the nature of the binding medium. In particular, in all samples characterized by the formation of zinc carboxylates or more generally zinc complexes (those based on linseed oil and mastic as the binding medium), we detected a net decrease of the GL emission decay time. This finding suggests that the GL emission is affected by the formation of zinc complexes. Hermans et al. [14,16] demonstrated that the interaction between pigment and oil binder causes the migration of Zn^2+^ ions from the ZnO pigment particles to the carboxylic groups that are part of polymeric network, leading over time to the crystalline zinc soap aggregates. On the basis of this model, it is clear that the densities of zinc vacancies inside the zinc white crystal structure increase following the formation of zinc carboxylates, giving rise to a modification of the GL emission. Moreover, the observed decrease in lifetime of the GL emission decay kinetic may be associated with the formation of zinc vacancies at pigment–binder boundary. This hypothesis is further encouraged by the recent studies in which the green emission in zinc oxide nanostructures has been ascribed to surface zinc vacancies [20].

## 5. Conclusions

Here, the photoluminescence properties of zinc white mixed with different organic binders were studied, highlighting two distinct emissions via trap state levels related to distinct types of defects within the ZnO crystalline structure. In particular, we report a TS emission in the blue spectral region, which has heretofore received little attention, and the well-known green emission. A better understanding of the peculiar nature of these TS emissions could be elucidated in the future by complementary information from theoretical simulations of zinc white paints and experimental spectroscopy studies, based for example on electronic paramagnetic spectroscopy (EPR) and hard X-ray photoelectron spectroscopy (HAXPES).

More interesting for conservative, both emissions are enhanced or modified following interaction of the metal pigment with the surrounding organic environment. The decrease of the decay kinetic of the green emission is correlated with the migration of Zn^2+^ from the semiconductor pigment and the consequent formation of zinc carboxylates, while the concentration of defects related to the blue emission seems to be highly affected by the active groups of binder. These changes could be used as quick indicators of the initial formation of carboxylates in zinc paints and the pigment–binder interaction that causes degradation. For this purpose, the study will be extended in the future to artificially aged samples, historical paints and microsamples. The same approach could be helpful for studying other inorganic paints often subjected to degradation phenomena, including weakly emitting lead white and strongly emitting cadmium-based paints.

## Figures and Tables

**Table 1:** Analyzed samples of zinc white and of reference materials.

Paint Mock Ups	Reference Samples
Zinc white + linseed oil (ZWLSO)	Zinc oxide (analytical grade) (ZNO)
Zinc white + mastic (ZWM)	Zinc white powder (ZW)
Zinc white + gum arabic (ZWGA)	Linseed oil (LSO)
Zinc oxide + linseed oil (ZNOLSO)	Mastic (M)
Zinc oxide + mastic (ZNOM)	Gum arabic (GA)
Zinc oxide + gum arabic (ZNOGA)	Zinc stereate (ZNS)
Commerical zinc white oil paint (ZWT)	Zinc palmitate (ZNP)

**Table 2:** Summary of Fourier transform infrared spectroscopy (FTIR) bands and assignments related to binding media and complex formation in different zinc white mock-up samples.

Sample	Band/cm^−1^	Assignment	Attribution	Reference
Zinc white (ZW)	3390	ν O-H	Hydrated zinc carbonate zinc carbonate	[13]
	1550	CO_3_(ν_3_)		
	1420	CO_3_		
	1510	CO_3_		
	1380	CO_3_		
	830	CO_3_ (ν_2_)		
Zinc white in linseed oil (ZWLSO)	3400	ν O-H	Linseed oil	[11,12]
	2920	ν_s_ CH_2_		
	2850	ν_as_CH_2_		
	1740	ν C=O ester		
	1460	δ CH_2_		
	1170	ν C-O-C ester		
	1598	sh COO^−^	Carboxylate formation	[11,12,16]
	1550	ν_as_ COO^−^		
	1410	ν_s_ COO^−^		
Zinc white in mastic (ZWM)	1587	ν COO^−^	Carboxylate complex	[23,29]
Zinc white in gum arabic (ZWGA)	3370	ν O-H	Gum arabic	[11,12]
	2930	ν_s_ CH_2_		
	1600	ν_s_ COO^−^	Organic acids in gum arabic	
	1420	δ CH_2_	Gum arabic	
	1070	ν_as_ C-O-C ether (ring)		
Zinc white commercial paint (ZWT)	1585	ν COO^−^	Amorphous zinc carboxylates	[16]
Zinc palmitate (ZNP)	1700	ν C=O	Unreacted palmitic acid	[28]
	1540	ν_as_ COO^−^	Carboxylate formation	[11,12]
	1400	ν_s_ COO^−^		
Zinc staerate (ZNS)	1700	ν C=O	Unreacted staeric acid	[29]
	1530	ν_as_ COO^−^	Carboxylate formation	[11,12]
	1400	ν_s_ COO^−^		

**Table 3:** Ratio between the trap state levels (TS) and the band-to-band recombination (BE) emission intensity in mock-up samples with respect to the same ratio in pure zinc white pigment (sample ZW). The ratio was performed considering emission as counts per sec in the following selected spectral bands: 370–400 nm, 410–450 nm and 500–550 nm for the band-to-band (BE), blue (BL) and green (GL) emission respectively.

Samples	(BL/BE)_sample_/(BL/BE)_ZW_	(GL/BE)_sample_/(GL/BE)_ZW_
ZNO	1.2	1.1
ZW	1.0	1.0
ZWLSO	8.3	4.0
ZWM	4.8	2.1
ZWGA	10.3	2.3
ZWT	2.9	3.4

## Supplementary Materials

The following are available online at www.mdpi.com/1996-1944/10/4/340/s1. Figure S1: FTIR spectrum of pure ZnO and ZnO mock-ups. Figure S2: TRPL datasets of zinc white and zinc white paint mock-ups made with different binders. Figure S3: TRPL datasets of zinc oxide and zinc oxide paint mock-ups made with different binders. Figure S4: TRPL datasets of the commercial zinc white paint. Figure S5: BE emission decay kinetic of all zinc oxide- and zinc white-based samples. Table S1: results of non-linear fit of BE emission decay kinetic. Figure S6: TS emission decay kinetic of all zinc oxide- and zinc white-based samples. Table S2: results of non-linear fit of TS emission decay kinetic. Figure S7: PL emission spectrum and PL decay kinetic of reference binders. Figure S8: PL emission spectrum and PL decay kinetic of zinc palmitate and zinc stereate.

## References

[1] Pelant I., Valenta J. (2012). Luminescence Spectroscopy of Semiconductors.

[2] Chen Q., Zhou H., Song T., Luo S., Hong Z., Duan H., Dou L., Liu Y., Yang Y. (2014). Controllable Self-Induced Passivation of Hybrid Lead Iodide Perovskites toward High Performance Solar Cells. Nano Lett..

[3] Narukawa Y., Kawakami Y., Fujita S., Fujita S. (1997). Recombination dynamics of localized excitons in In0.20Ga0.80N-In0.05Ga0.95 multiple quantum wells. Phys. Rev. B.

[4] Knowles K.E., McArthur E.A., Weiss E.A. (2011). A Multi-Timescale Map of Radiative and Non-radiative Decay Pathways for Excitons in CdSe Quantum Dots. ACS Nano.

[5] Gorgis A., Flissikowski T., Brandt O., Chèze C., Geelhaar L., Riechert H., Grahn H.T. (2012). Time-resolved photoluminescence spectroscopy of individual GaN nanowires. Phys. Rev. B.

[6] Osmond G. (2012). Zinc white: A review of zinc oxide pigment properties and implications for stability in oil-based paintings. AICCM Bull..

[7] Eastaugh N., Walsh V., Chaplin T. (2007). Pigment Compendium: A Dictionary of Historical Pigments.

[8] Jacobsen A.E. (1941). Zinc soaps in paints. Ind. Eng. Ind..

[9] Van Loon A., Noble P., Boon J. White Hazes and Surface Crusts in Rembrandt’s Homer and Related Paintings. Proceedings of the 16th triennial conference ICOM-CC 2011.

[10] Keune K., Mass J., Mehta A., Church J., Meirer F. (2016). Analytical imaging studies of the migration of degraded orpiment, realgar, and emerald green pigments in historic paintings and related conservation issues. Herit. Sci..

[11] Robinet L., Corbeil M.-C. (2003). The Characterization of Metal Soaps. Stud. Conserv..

[12] Brambilla L., Riedo C., Baraldi C., Nevin A., Gamberini M.C., D’Andrea C., Chiantore O., Goidanich S., Toniolo L. (2011). Characterization of fresh and aged natural ingredients used in historical ointments by molecular spectroscopic techniques: IR, Raman and fluorescence. Anal. Bioanal. Chem..

[13] Clementi C., Rosi F., Romani A., Vivani R., Brunetti B.G., Miliani C. (2012). Photoluminescence properties of zinc oxide in paints: a study of the effect of self-absorption and passivation. Appl. Spectrosc..

[14] Hermans J.J., Keune K., van Loon A., Corkery R.W., Iedema P.D. (2016). Ionomer-like structure in mature oil paint binding media. RSC Adv..

[15] Kryven I., Duivenvoorden J., Hermans J., Iedema P.D. (2016). Random Graph Approach to Multifunctional Molecular Networks. Macromol. Theory Simul..

[16] Hermans J.J., Keune K., van Loon A., Iedema P.D. (2016). The crystallization of metal soaps and fatty acids in oil paint model systems. Phys. Chem. Chem. Phys..

[17] Rodnyi P.A., Khodyuk I.V. (2011). Optical and luminescence properties of zinc oxide (Review). Opt. Spectrosc..

[18] Umit O., Daniel H., Hadis M. (2010). ZnO devices and applications: A review of current status and future prospects. Proc. IEEE.

[19] Wang Z.G., Zu X.T., Zhu S., Wang L.M. (2006). Green luminescence originates from surface defects in ZnO nanoparticles. Phys. E Low-Dimens. Syst. Nanostruct..

[20] Fabbri F., Villani M., Catellani A., Calzolari A., Cicero G., Calestani D., Calestani G., Zappettini A., Dierre B., Sekiguchi T. (2014). Zn vacancy induced green luminescence on non-polar surfaces in ZnO nanostructures. Sci. Rep. (Nat. Publ. Group).

[21] Artesani A., Bellei S., Capogrosso V., Cesaratto A., Mosca S., Nevin A., Valentini G., Comelli D. (2016). Photoluminescence properties of zinc white: An insight into its emission mechanisms through the study of historical artist materials. Appl. Phys. A.

[22] Comelli D., Nevin A., Brambilla A., Osticioli I., Valentini G., Toniolo L., Fratelli M., Cubeddu R. (2012). On the Discovery of an Unusual Luminescent Pigment in Van Gogh’s Painting “Les bretonnes et le pardon de pont Aven”. Appl. Phys. A.

[23] Seoane E. (1956). Further crystalline constituents of gum mastic. J. Chem. Soc. (Resumed).

[24] Gunn M., Chottard G., Rivière E., Girerd J.-J., Chottard J.-C. (2002). Chemical Reactions between Copper Pigments and Oleoresinous Media. Stud. Conserv..

[25] Poli T., Piccirillo A., Zoccali A., Conti C., Nervo M., Chiantore O. (2014). The role of zinc white pigment on the degradation of shellac resin in artworks. Polym. Degrad. Stab..

[26] Doménech-Carbó M.T., Kuckova S., de la Cruz-Cañizares J., Osete-Cortina L. (2006). Study of the influencing effect of pigments on the photoageing of terpenoid resins used as pictorial media. J. Chromatogr. A.

[27] Bonaduce I., Brecoulaki H., Colombini M.P., Lluveras A., Restivo V., Ribechini E. (2007). Gas chromatographic–mass spectrometric characterisation of plant gums in samples from painted works of art. J. Chromatogr. A.

[28] Hermans J.J., Keune K., van Loon A., Iedema P.D. (2015). An infrared spectroscopic study of the nature of zinc carboxylates in oil paintings. J. Anal. At. Spectrom..

[29] Mazzeo R., Prati S., Quaranta M., Joseph E., Kendix E., Galeotti M. (2008). Attenuated total reflection micro FTIR characterisation of pigment–binder interaction in reconstructed paint films. Anal. Bioanal. Chem..

[30] Osmond G., Boon J.J., Puskar L., Drennan J. (2012). Metal Stearate Distributions in Modern Artists' Oil Paints: Surface and Cross-Sectional Investigation of Reference Paint Films Using Conventional and Synchrotron Infrared Microspectroscopy. Appl. Spectrosc..

[31] Shao X., Zhang J. (2008). A simple preparation technique of ZnO thin film with high crystallinity and UV luminescence intensity. J. Phys. Chem. Solids.

[32] Thoury M., Echard J.-P., Réfrégiers M., Berrie B., Nevin A., Jamme F., Bertrand L. (2011). Synchrotron UV−Visible Multispectral Luminescence Microimaging of Historical Samples. Anal. Chem..

[33] Bertrand L., Réfrégiers M., Berrie B., Échard J.P., Thoury M.A. (2013). A multiscalar photoluminescence approach to discriminate among semiconducting historical zinc white pigments. Analyst.

[34] Anžlovar A., Marinšekb M., Orela Z.C., Žigona M. (2015). Basic zinc carbonate as a precursor in the solvothermal synthesis of nano-zinc oxide. Mater. Des..

[35] Zeng H., Duan G., Yang S., Xu X., Cai W. (2010). Blue luminescence of ZnO nanoparticles based on non-equilibrium processes: Defect origins and emission controls. Adv. Funct. Mater..

[36] Mishra S.K., Srivastava R.K., Prakash S.G., Yadav R.S., Panday A.C. (2010). Photoluminescence and photoconductive characteristics of hydrothermally synthesized ZnO nanoparticles. Opto-Electron. Rev..

[37] Chen H., Gu S., Tang K., Zhu S., Zhu Z., Ye J., Zhang R., Zheng Y. (2011). Origins of green band emission in high-temperature annealed N-doped ZnO. J. Lumin..

