# Supplementary material for: A Photoluminescence Study of the Changes Induced in the Zinc White Pigment by Formation of Zinc Complexes
Source: Materials (Basel). 2017 Mar 25;10(4):340. doi: 10.3390/ma10040340 (PMC5506911; doi:10.3390/ma10040340)
Supplement: Supplementary file 1 [file materials-10-00340-s001.pdf]

Supplementary Material

In the following, samples will be labeled as reported in Table 1 in the main manuscript.

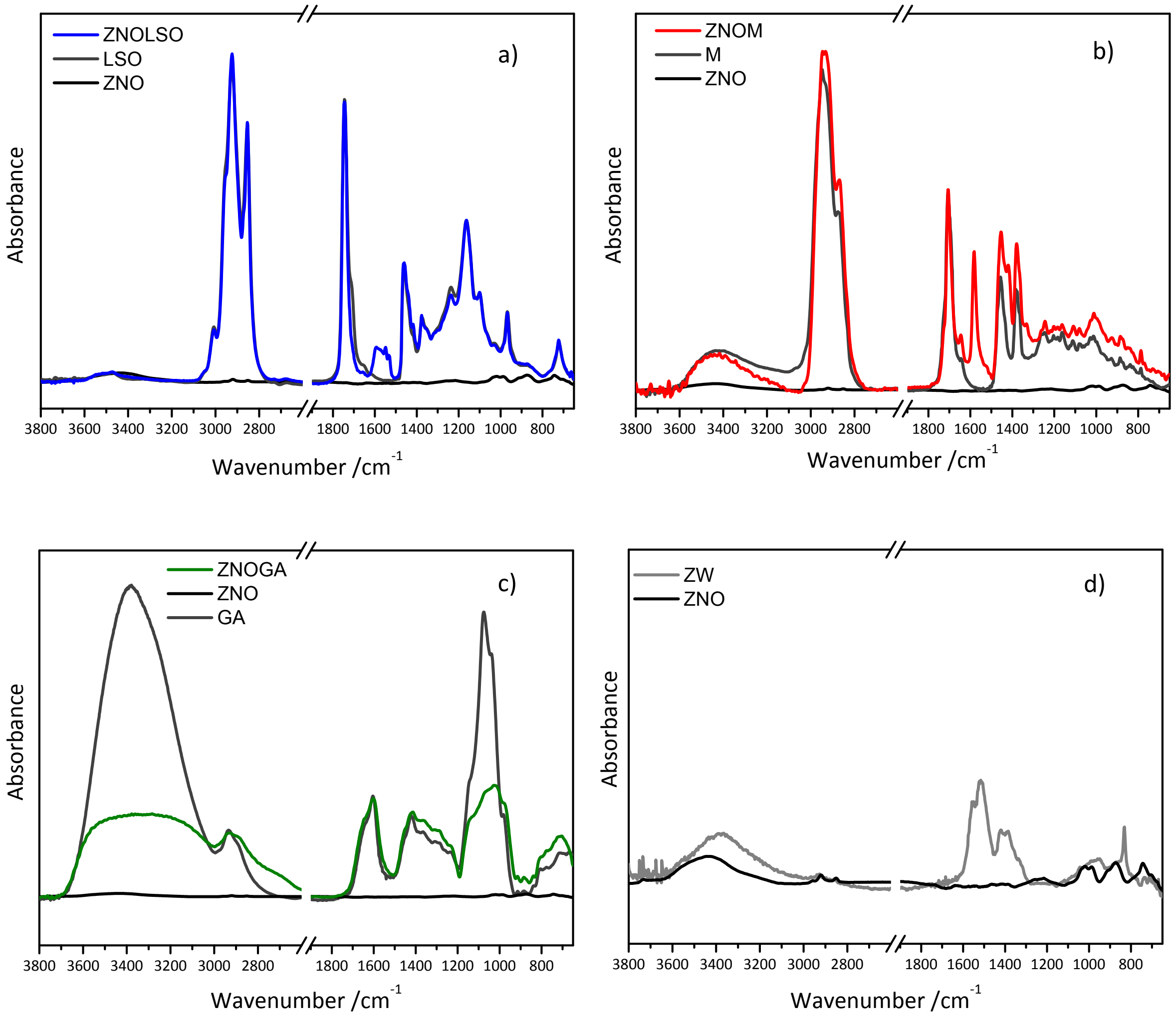

Figure S1. FTIR spectrum of (**a**) zinc oxide powder (ZNO), linseed oil (LSO) and paint based on zinc oxide and stand linseed oil (ZNOLSO). (**b**) zinc oxide powder (ZNO), mastic (M) and paint based on zinc oxide and mastic (ZNOM). (**c**) zinc oxide powder (ZNO), gum arabic (GA) and paint based on zinc oxide and gum arabic (ZNOGA). (**d**) zinc white (ZW) and zinc oxide (ZNO) powder. Spectra are shown following background correction.

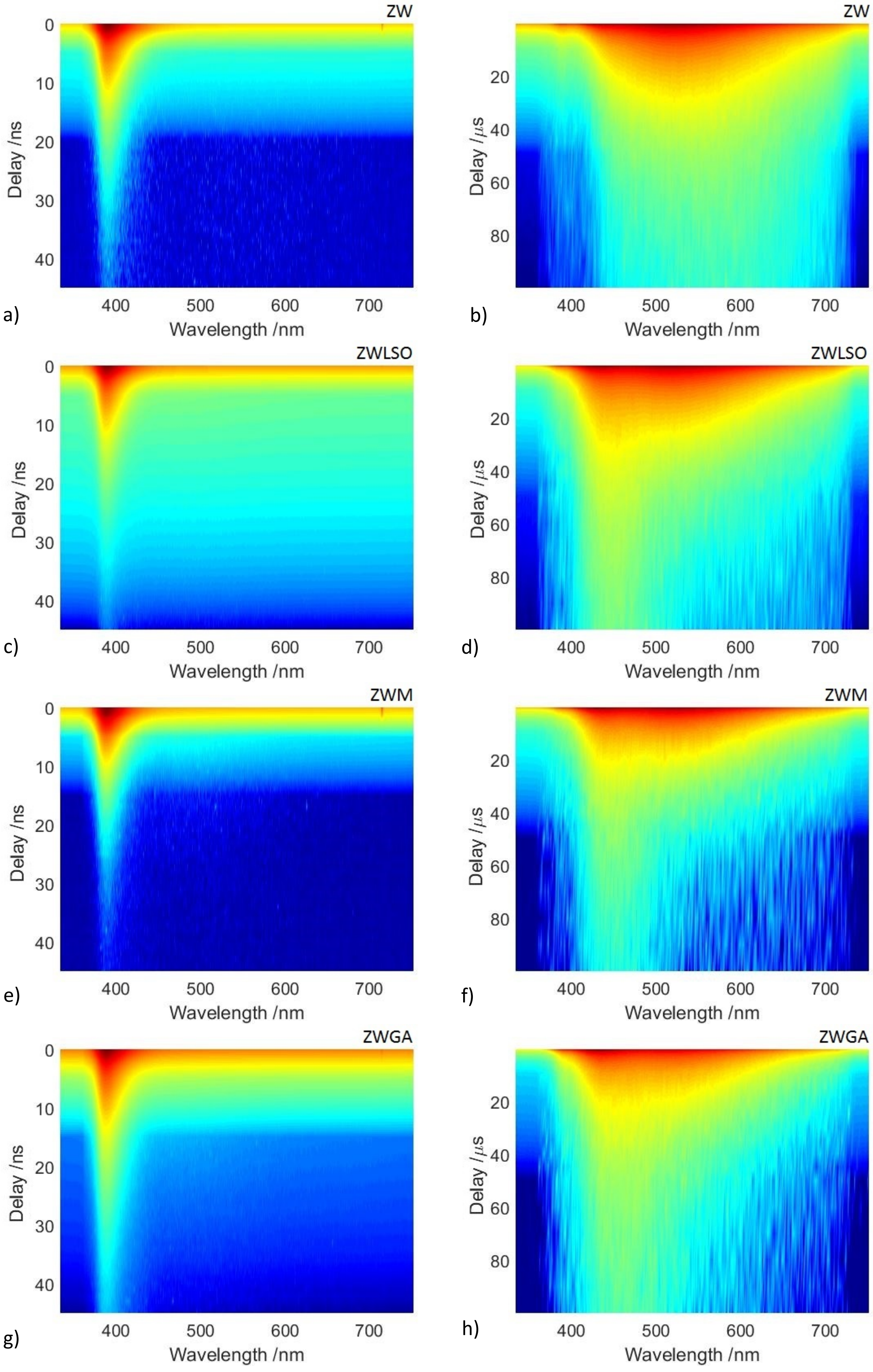

Figure S2. Two-dimensional TRPL datasets, taken as a function of emission wavelength and delay time of gating detection respect to laser excitation for pure zinc white powder (ZW) and zinc white paint mock-ups (ZWLSO, ZWM and ZWGA). **a,c,e,g)** the photoluminescence at nanosecond delay that allows to detect band-to-band (BE) mission. **b,d,f,h)** the photoluminescence at microsecond delay that allows to detect the trap state (TS) emissions.

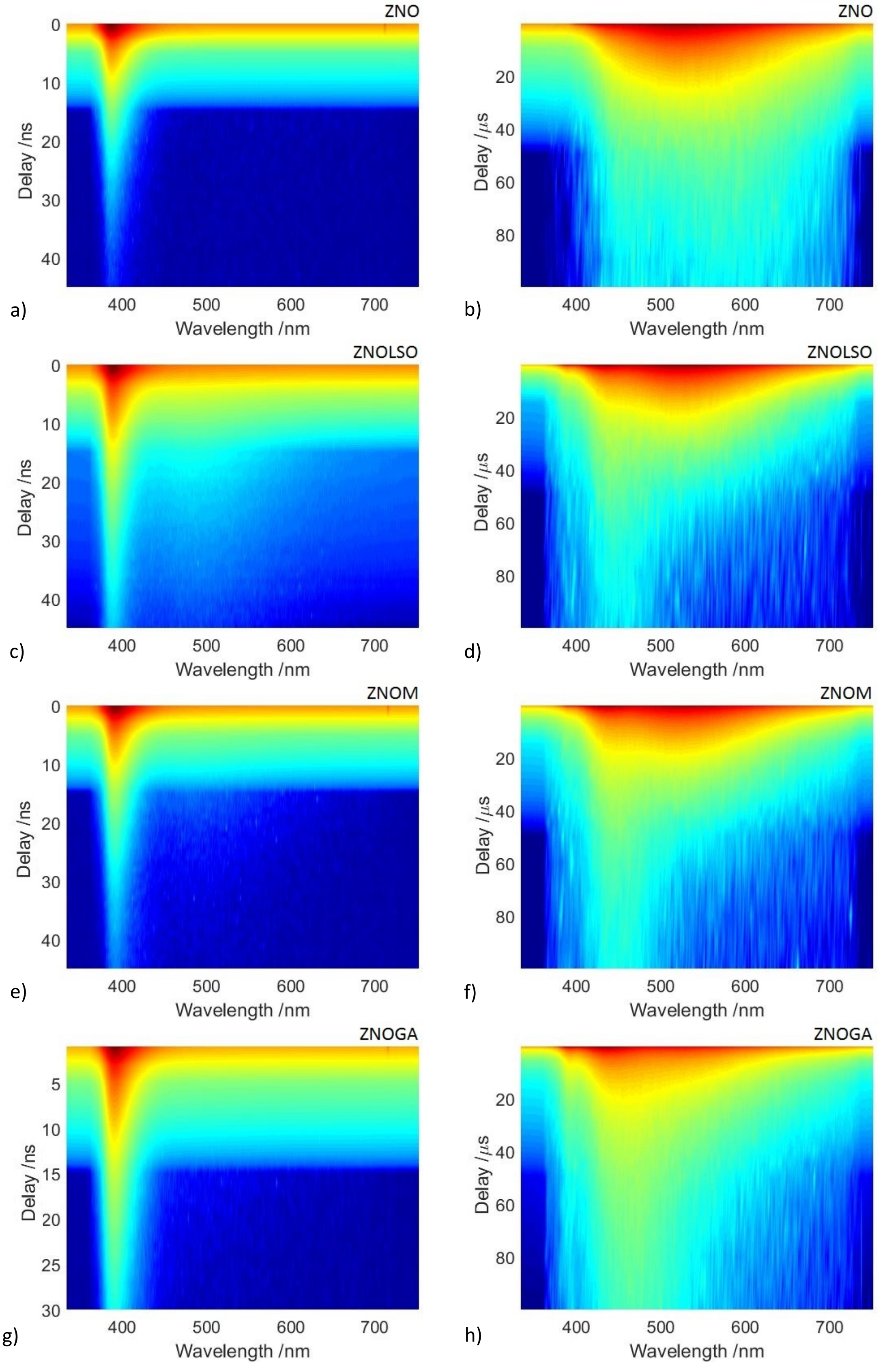

Figure S3. Two-dimensional TRPL datasets, taken as a function of emission wavelength and delay time of gating detection respect to laser excitation for pure zinc oxide powder (ZNO) and zinc oxide paint mock-ups (ZNOLSO, ZNOM and ZNOGA). **a,c,e,g)** the photoluminescence at nanosecond delay that allows to detect band-to-band (BE) emission. **b,d,f,h)** the photoluminescence at microsecond delay that allows to detect the trap state (TS) emissions.

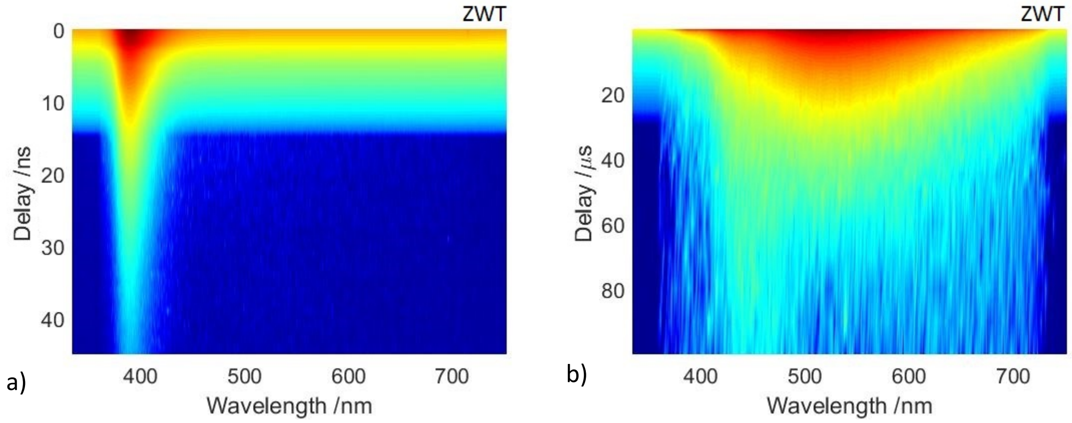

Figure S4. Two-dimensional TRPL datasets, taken as a function of emission wavelength and delay time of gating detection respect to laser excitation for the zinc white commercial paint. **a)** The photoluminescence at nanosecond delay that allows to detect band-to-band (BE) emission. **b)** The photoluminescence at microsecond delay that allows to detect the trap state (TS) emissions.

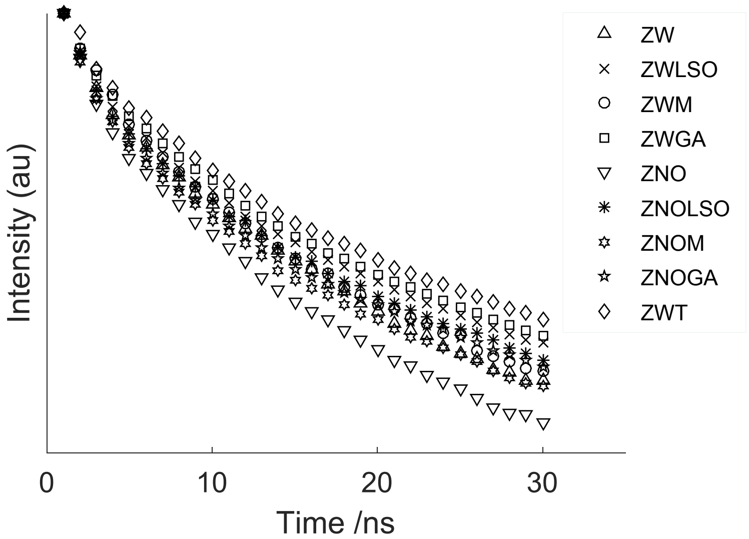

Figure S5. Band-to-band (BE) emission decay kinetic, as a function of delay time respect to pulsed excitation, detected in the spectral band 370-410 nm. The intensity decay kinetic for each paint mock-up and reference material is illustrated with different symbol, explicated in the legends.

Table S1. Results of analysis of band-to-band (BE) emission decay kinetic fitted with a three-exponential decay model. Here the lifetime (τ_i) and the relative weight (A_i%) are reported. Values are given for all studied samples for the BE emission at 370-410 nm.

	A1 (%)	A2 (%)	A3 (%)	τ1 [ns]	τ2 [ns]	τ3 [ns]	R-square
ZW	0.1	3.8	96.1	9.6	3.5	0.9	0.999
ZWLSO	0.3	5.1	94.6	8.3	2.9	0.8	1.000
ZWM	0.4	8.7	90.9	8.0	3.2	1.3	0.997
ZWGA	0.3	8.6	91.0	9.1	3.1	0.9	1.000
ZNO	0.0	2.4	97.6	9.0	3.1	0.8	0.998
ZNOLSO	0.1	3.4	96.5	8.6	2.8	0.8	0.998
ZNOM	0.1	3.1	96.8	7.9	2.8	0.7	0.999
ZNOGA	0.3	10.2	89.5	10.1	3.2	0.9	0.999
ZWT	0.3	89.5	10.2	12.0	1.2	3.8	0.993

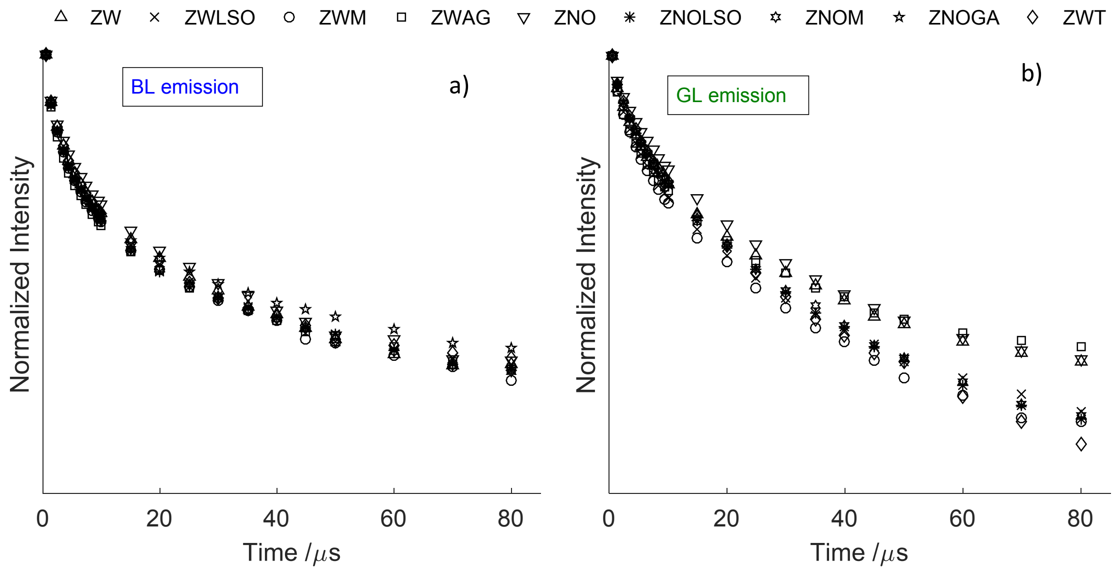

Figure S6. Trap state (TS) emission decay kinetic, as a function of delay time respect to pulsed excitation t = 0.5 μs has been set as time zero. **a)** decay of blue emission (BL, 410-450 nm); **b)** decay of green emission (GL, 500-550 nm). In each chart, the intensity decay for each paint mock-up and reference are illustrated with different symbol, explicated in the legends.

Table S2. Results of analysis of trap state (TS) emission decay kinetic fitted with a three exponential model. Here the lifetime (τ_i) and the relative weight (A_i%) are reported. Values are given for all studied samples for the TS emission in two spectral bands: the blue band (BL) at 410-450 nm and the green band (GL) at 500-550 nm.

	BL (410-450 nm)						
	**A1 (%)**	**A2 (%)**	**A3 (%)**	**τ1 [μs]**	**τ2 [μs]**	**τ3 [μs]**	**R-square**
ZW	1.1	87.6	11.4	18.3	0.6	3.0	0.998
ZWLSO	0.8	89.6	9.5	22.1	0.8	3.6	0.999
ZWM	0.8	88.5	10.7	22.8	0.7	3.4	0.998
ZWGA	0.7	90.9	8.5	23.8	0.6	3.4	0.998
ZNO	1.5	85.8	12.8	18.4	0.6	3.3	0.998
ZNOLSO	0.8	88.6	10.6	24.7	0.7	3.6	0.999
ZNOM	0.8	90.4	8.9	21.8	0.7	3.4	0.999
ZNOGA	0.9	87.9	11.2	24.9	0.7	3.5	0.999
ZWT	0.9	87.9	11.2	22.6	0.7	3.7	0.998
	**GL (500-550 nm)**						
	**A1 (%)**	**A2 (%)**	**A3 (%)**	**τ1 [μs]**	**τ2 [μs]**	**τ3 [μs]**	**R-square**
ZW	2.6	76.8	20.7	15.7	0.9	3.9	0.999
ZWLSO	1.5	81.9	16.6	15.8	1.0	4.3	0.999
ZWM	1.7	79.3	19.0	15.1	0.9	3.9	0.999
ZWGA	1.8	81.9	16.3	21.7	0.9	4.4	0.999
ZNO	3.7	66.5	29.8	18.0	1.1	4.8	0.999
ZNOLSO	3.1	71.7	25.2	14.3	1.1	4.2	1.000
ZNOM	2.7	80.4	16.9	14.6	1.1	4.4	1.000
ZNOGA	2.3	73.1	24.6	24.8	0.9	4.6	0.999
ZWT	2.3	73.1	24.6	15.1	1.2	4.9	0.999

Reference Samples

The PL emission spectrum and decay kinetic of reference samples are here reported. It is here noted that all the analyzed reference samples do not show any detectable PL emission at the microsecond time scale. Hence, the detected PL emission (reported in the following figures) partially overlaps with the BE emission of zinc white, but does not give rise to any superposition with the microsecond TS emission of zinc white.

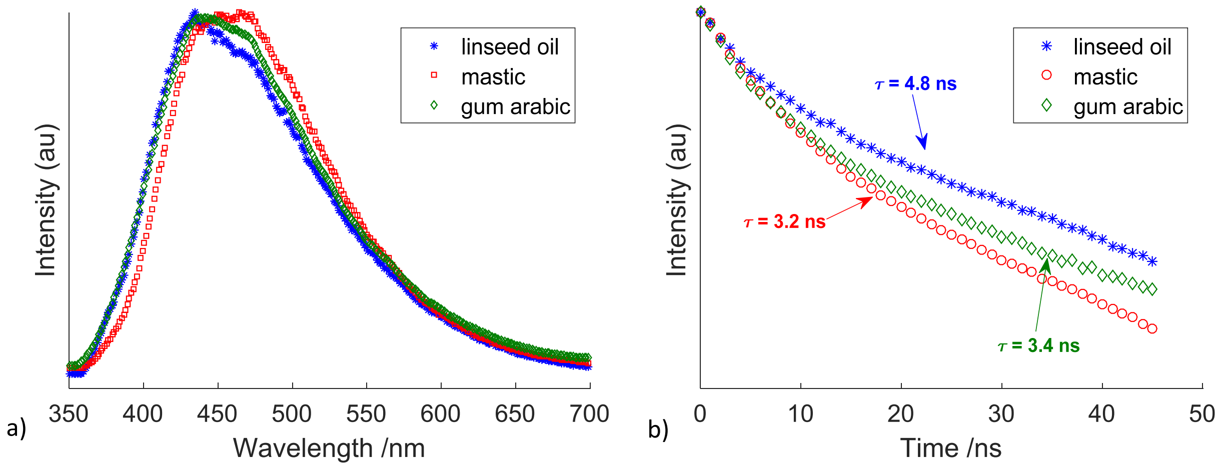

Figure S7. a) PL gated spectrum (time delay: 1-5 μs) of binders: linseed oil, mastic and gum arabic. All spectra are normalized and corrected for the instrumental efficiency. **b**) PL emission decay kinetic, as a function of delay time respect to pulsed excitation, in the spectral band 400-550 nm. The PL spectrum and decay kinetic for each reference binder are illustrated with different symbol, explicated in the legend.

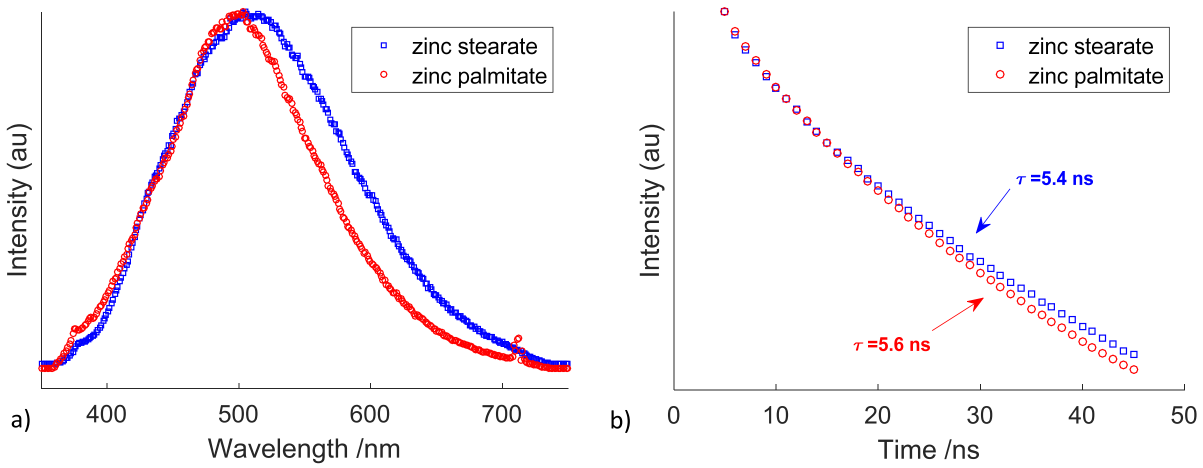

Figure S8 a) PL gated spectrum (time delay: 1-5 μs) of zinc stearate (ZNS) and zinc palmitate (ZNP). All spectra are normalized and corrected for the instrumental efficiency. **b**) PL emission decay kinetic, as a function of delay time respect to pulsed excitation, in the spectral band 450-600 nm. The PL spectrum and decay kinetic for each reference material are illustrated with different symbol, explicated in the legend.
